# CADM2, as a new target of miR-10b, promotes tumor metastasis through FAK/AKT pathway in hepatocellular carcinoma

**DOI:** 10.1186/s13046-018-0699-1

**Published:** 2018-03-05

**Authors:** Dongliang Li, Yongjian Zhang, He Zhang, Chao Zhan, Xin Li, Tu Ba, Zini Qiu, Fang E, Guixiang Lv, Chendan Zou, Chuxuan Wang, Lining Si, Chaoxia Zou, Qiang Li, Xu Gao

**Affiliations:** 1 Department of Biochemistry and Molecular Biology, Harbin Medical University, Harbin, Heilongjiang 150081 China; 2 Department of Hepatobiliary and Pancreas, Heilongjiang Cancer Hospital, Harbin, China; 3 Department of General Surgery, the Second Affiliated Hospital of Harbin Medical University, Harbin, China; 4 Translational Medicine Research and Cooperation Center of Northern China, Heilongjiang Academy of Medicine Sciences, Harbin, Heilongjiang 150081 China; 5 Key Laboratory of Cardiovascular Medicine Research of Harbin Medical University, Ministry of Education, Harbin, China; 6 Department of Critical-care Medicine, the Affiliated Hospital of Qinghai University, Xining, Qinghai China; 7 Department of Respiratory Medical Oncology, Heilongjiang Cancer Hospital, Harbin, China; 8 Department of Neck and Breast Surgery, Mudanjiang Tumor Hospital, Mudanjiang, China

**Keywords:** Hepatocellular carcinoma, Cell adhesion molecule 2, MicroRNA-10b, Tumor metastasis, Epithelial-mesenchymal transition

## Abstract

**Background:**

Cell adhesion molecules (CADMs) comprise of a protein family whose functions include maintenance of cell polarity and tumor suppression. Hypo-expression of CADM2 gene expression has been observed in several cancers including hepatocellular carcinoma (HCC). However, the role and mechanisms of CADM2 in HCC remain unclear.

**Methods:**

The expression of CADM2 and miRNA-10b (miR-10b) in HCC tissues and cell lines were detected using real-time PCR and Western blotting. Immunofluorescence was used to detect Epithelial-mesenchymal transition (EMT) progression in HCC cell lines. Dual-luciferase reporter assay was used to determine miR-10b binding to CADM2 3’UTR. Wound healing assay and Transwell assay were performed to examine the migration and invasion of HCC cells.

**Results:**

We report the effect of CADM2 as a tumor suppressor in HCC. Firstly, we confirmed that CADM2 expression was significantly down regulated in HCC tissues compared to normal tissues according to TCGA data analysis and fresh HCC sample detection. Secondly, overexpression of CADM2 could inhibit EMT process, migratory and invasion ability of HCC cells. Furthermore, the results indicated that CADM2 is a direct target of miR-10b in HCC cells and miR-10b/CADM2 modulates EMT process and migration ability via focal adhesion kinase (FAK) /AKT signaling pathway in HCC.

**Conclusions:**

Our study demonstrates that miR-10b-CADM2-FAK/AKT axis plays an important role in HCC metastasis, which might be a novel potential therapeutic option for HCC treatment.

**Electronic supplementary material:**

The online version of this article (10.1186/s13046-018-0699-1) contains supplementary material, which is available to authorized users.

## Background

Hepatocellular Carcinoma (HCC) is the second cause of cancer death following lung cancer, and the sixth most common cancer worldwide, with China’s annual HCC mortality rate accounting for approximately 55% globally [1, 2]. The two main reasons why the prognosis of HCC patients after radical surgery is poor are cancer recurrence and metastasis [3]. Although various therapies and treatments have been rapidly developing over the years, no effective treatments for primary liver cancer metastasis has been developed [3, 4]. Thus, it is necessary to uncover the underlying mechanisms that drive metastasis so as to establish a better treatment approach for HCC.

The tumor metastasis is a multistep process that results in the formation of new foci away from the primary focus [5, 6]. Epithelial-mesenchymal transition (EMT) is the process that epithelial cells transdifferentiate into mesenchymal cells. EMT acts as the foundation on which cancer cells detach themselves from the peripheral cells and form their new metastases through infiltration of the blood or lymphatic circulation system [7]. Various studies have shown that EMT is a dynamic cell activity that plays an essential role in tumor metastasis [8, 9].

Cell adhesion molecules (CADMs) belong to an immunoglobulin superfamily [10]. Multiple normal tissues express CADMs. However, a variety of cancerous tissues either lack CADMs or express them at reduced levels. Recent studies suggest that CADMs might serve as tumor suppressors. For example, CADM1 has been reported to be reduced in lung cancer [11], prostate cancer [12], esophageal cancer [13], and breast cancer [14], and several articles have reported that CADM3 and CADM4 also functions as tumor suppressor in various types of cancer cells [15–17]. CADM2 is the last one that is reported as tumor suppressor gene in CADMs family. Previous studies report that CADM2 acts as a tumor suppressor in prostate cancer and renal cell carcinoma progression [18, 19]. Yang et al. find that low CADM2 expression predicts high recurrence risk of HCC patients after hepatectomy [20]. What’s more, the data from GEO (Gene Expression Omnibus) database also indicate that the expression level of CADM2 in liver cancer with venous metastasis is apparently lower than that in those not transferred in the vein [21]. However, the role and mechanism of CAMD2 in HCC remains unclear.

In this study, we identified that overexpression of CADM2 restrained EMT in HCC cells, thereby influencing migration and invasion of HCC cells. Most importantly, we found out that CADM2 is a direct target gene of miR-10b in HCC cells and upregulation of miR-10b results in the decrease of CADM2 expression in turns promotes EMT progression. The influence of CADM2 on EMT of liver cancer cells is further studied through FAK/AKT pathway.

## Methods

### Patients and clinical samples

From January 2017 to October 2017, 36 fresh primary tumor samples and their corresponding, non-tumorous tissues were obtained from hepatic carcinoma patients at Heilongjiang Cancer Hospital (Harbin, China). Consents from each patient and approval by the local ethics committee were obtained (for the characteristics of all patients, see Additional file 1: Table S1). All specimens were histopathologically confirmed. HCC was graded according to the World Health Organization grading system and staged according to the American Joint Committee on Cancer (AJCC) tumor node-metastasis (TNM) staging system.

### Cell culture and reagents

Human normal liver cell line HL7702 and HCC cell lines HepG2, Huh-7, Hep3B were maintained in Dulbecco’s modified Eagle’s medium (DMEM) supplemented with 10% fetal bovine serum (FBS, GIBCO, Carlsbad, CA, USA), 100 U/ml penicillin G and 100 μg/ml streptomycin at 37 °C in a humidified incubator containing 5% CO_2_. LY294002 (#HY-10108, 2-morpholin-4-yl-8-phenylchromen-4-one) was purchased from MedChem Express (MCE, USA). 5 mg/ml LY294002 (storage solution) was prepared using Dimethyl Sulfoxide (DMSO).

### Oligonucleotide synthesis and transfection

MiR-10b mimics and miR-10b inhibitors as well as their corresponding negative control were purchased from GenePharma (Shanghai, China). HepG2,Huh7 and Hep3B cells were transfected with miR-10b mimic or miR-10b inhibitor using Lipofectamine 2000 transfection reagent (Invitrogen, Carlsbad, CA) according to reagent protocols. The oligonucleotide sequences utilized in transfection had been listed in Additional file 2: Table S3. Plasmid pEZ-M98-CADM2 (pEX-CADM2) and its corresponding empty vector (pEX-NC) were purchased from GeneCopoeia (GeneCopoeia, USA). To overexpress CADM2 in HCC cell lines, pEX-CADM2 or pEX-NC was transfected using the method described previously [22]. Co-transfection of miR-10b mimic and pEX-CADM2 was conducted using Lipofectamine 2000 Reagent (Invitrogen, Carlsbad, CA).

### Real-time quantitative polymerase chain reaction (qRT-PCR)

Cultured cells were harvested and total RNA extracted by TRIzol Reagent (Life Technologies, USA). Then reverse transcription operated with ABI High Capacity cDNA reverse transcription Kit (Thermo Fisher Scientific, USA) as described previously [22]. The primers utilized in qRT-PCR had been listed in Additional file 2: Table S3.

### Western blotting analysis

Total protein was extracted using RIPA buffer and protein expression was analyzed by Western blotting as described previously [22]. GAPDH served as an endogenous control. The antibodies information utilized in Western blotting had been listed in Additional file 3: Table S4.

### Immunofluorescence analysis

Cells were rinsed with PBS and fixed with 4% paraformaldehyde for 30 min at room temperature followed by permeabilization with 0.1% sodium citrate plus 0.1% Triton X-100. The cells were subjected to immunofluorescent staining with primary antibody (Additional file 3: Table S4) for 16 h at 4 °C. Cells were then washed with cold PBS three times for 5 min each and incubated with fluorescence labeled secondary antibody (1:500, #ZF0511, ZSGB-BIO) for 30 min. The cells were visualized using inverted fluorescence microscope (FSX100, Olympus).

### Migration and invasion assay

A Transwell system (Corning Life Sciences) containing a polycarbonate filter (6.5 mm in diameter, 8 μm pore size) was used for migration and invasion assay. For cell invasion assay, the membrane undersurface was coated with 50 μl of matrigel mixed with DMEM at a 1:8 dilution and subsequently applied to the topside of the filter. By contrast, the filter was not coated for the cell migration assay. In both assays, cell suspensions (2 × 10^4^cells/well) were added to the upper chamber in medium without serum. Medium containing 1% FBS in the lower chamber served as a chemo-attractant. The cells that did not migrate or invade after 24 h of incubation were removed from the upper face of the filters by scrubbing with a cotton swab. Membranes were then fixed with 4% formaldehyde for 30 min at room temperature and stained with 0.1% crystal violet for 15 min. Finally, the number of migrating or invading cells was counted at × 200 magnification from ten different fields for each filter and analyzed to determine statistically significance.

A wound-healing assay was also applied to evaluate the cell migration ability. Cells were seeded in 3.5-cm plates and grown to a density of 70–80%. Then, a 200 μl pipette tip was used to create an artificial wound of scratched cells. The migrating distance was measured after 48 h or 72 h.

### Luciferase reporter assay

To construct a pmiR-CADM2–3′UTR plasmid containing the potential miR-10b binding sites, an 1168-bp sequence was amplified and inserted into the *Sac*I and *Xba*I sites of the pmir-GLO Dual Luciferase vector (Promega, Madison, WI, USA). This sequence contained the two predicted binding sites at 6767 nt–6775 nt and 7543 nt–7551 nt.

The plasmid with mutant-type (MUT1, the first binding site is mutated; MUT2, the second binding site is mutated) were inserted downstream of the luciferase of pmirGLO Dual-Luciferase vector. HEK293 cells were used to measure luciferase activity. When grown to 60–70% confluence, the cells were co-transfected with a 100 ng Luciferase plasmid along with a 60 pmol miR-10b mimic or NC mimic as described above. After incubation for 24 h at 37 °C, the luciferase activity was determined using the Dual Luciferase Reporter 1000 Assay System (Promega, Madison, WI, USA).

### TCGA data analysis

For mRNA expression analysis, the RNA-seq V2 data was obtained from TCGA (The Cancer Genome Atlas) database. The normalized RSEM value was extracted, log2 transformed and merged. Samples were grouped into cancerous and normal tissues based on barcodes. The mean, max, min and SD of each group were calculated and paired student’s *t* test was preformed to verify the statistical significance. For gene methylation analysis, the mean beat value was extracted from TCGA. Samples were matched base on barcodes. The *Pearson’*s correlation was calculated to test the link between CADM2 mRNA expression and methylation. The survival analysis was followed the method in Broad institute TCGA Genome Data Analysis Center. In brief, the normalized RSEM value and clinical data were obtained from TCGA database. Data was processed using R. Expression value of miR-10b was extracted and filtered. Samples that have low expression value (RSEM < 1) were excluded. The mean, median and SD were calculated. Patients were grouped based on miR-10b expression. High expressed group, in the group with high expression level, patients with a top 20% miR-10b expression level (*n* = 86). Low expressed group, patients with a low 20% miR-10b expression level (*n* = 87). The Kaplan-Meier plot was generated by “survival” package in R.

### Statistical analysis

SPSS was used for the statistical analysis. All values are expressed as the mean ± SEM, and all experiments were repeated at least three times. Student’s *t-*test was used to determine the statistical significance of the differences between groups. Comparative *t*-test was used for the clinical sample analysis. Differences with *P* < 0.05 were considered significant (*P < 0.05, **P < 0.01, ***P < 0.001).

## Results

### Hypo-expression of CADM2 in HCC tissues is related to poor prognosis

**Fig. 1:**
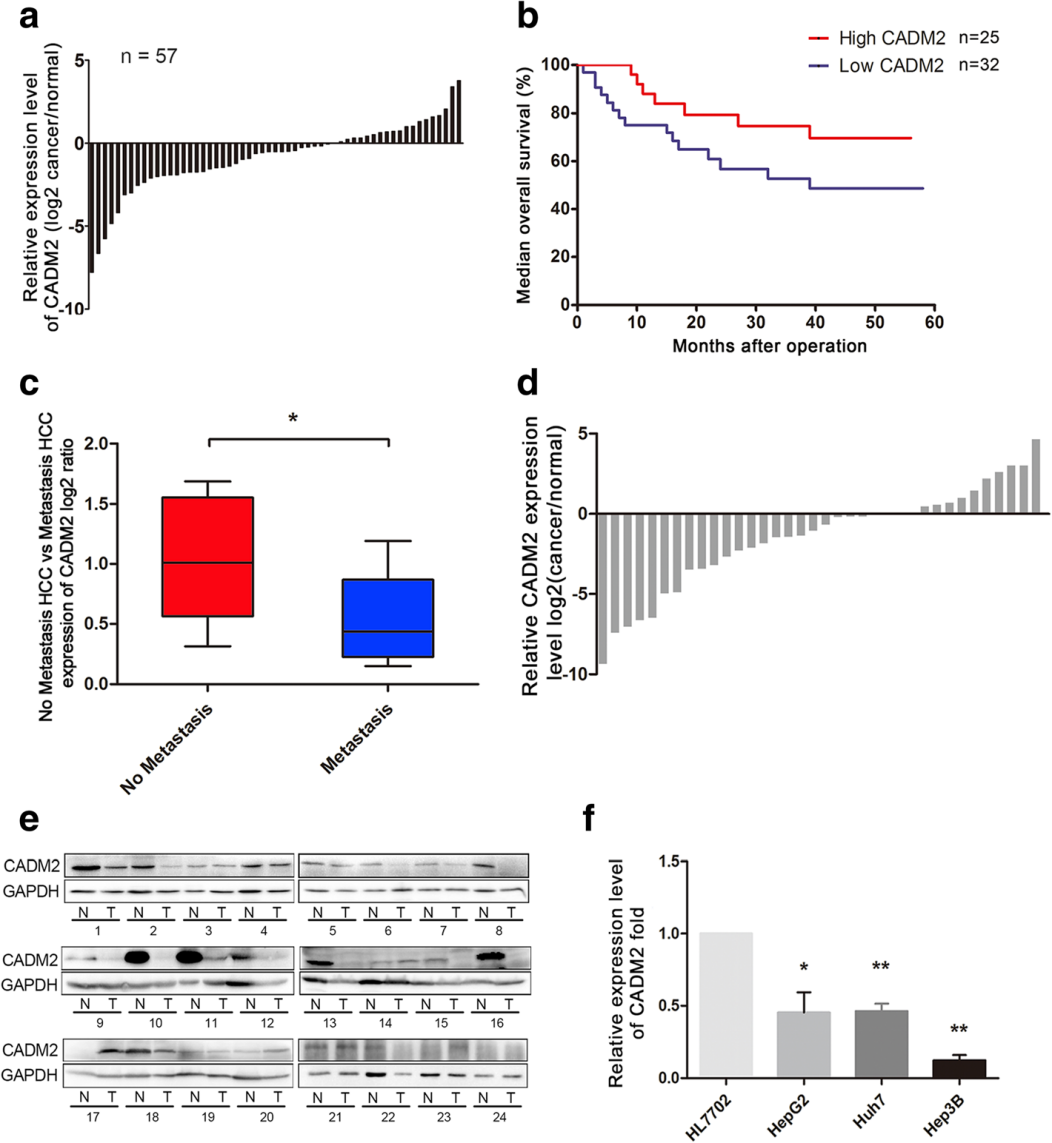
Lower expression of CADM2 was observed in HCC samples. **a** Analysis GEO data (GSE27150) of CADM2 expression levels in HCC and adjacent normal tissues (n = 57). **b** Kaplan-Meier analysis of median overall survival of patients with different CADM2 expression Kaplan-Meier analysis of median overall survival of patients with different CADM2 expression levels from GEO database (GSE27150, *P* = 0.0757, Red: High expression CADM2, Blue: Low expression CADM2). **c** Analysis GEO data (GDS3091) of CADM2 expression levels in HCC patients with venous metastasis (n = 9) and without venous metastasis (n = 11) from GEO data (GDS3091) were analyzed. **d** Relative CADM2 expression levels in HCC samples were detected by qRT-PCR. (n = 36, *P* = 0.0226). **e** CADM2 expression in HCC samples was examined using western blotting. GAPDH was used as a loading control. **f** qRT-PCR for detecting CADM2 mRNA expression levels in several liver cell lines as indicted. (*P < 0.05; **P < 0.01)

To investigate the expression level of CADM2 in HCC and its clinical significance, we analyzed the high-throughput data from the GEO Database (GSE27150, GDS3091) and it confirmed that CADM2 was significantly down regulated in HCC tissues in comparison with the matched normal samples (Fig. 1a). Furthermore, survival analysis indicated a strong correlation between CADM2 mRNA expression and overall survival of patients in dataset GSM27150. In other words, the lower expression of CADM2 mRNA was associated with poor prognosis (Fig. 1b). In addition, the expression of CADM2 in HCC tissues with and without venous metastasis from GEO was also analyzed. The expression of CADM2 in liver cancer samples with venous metastasis was significantly lower than those without venous metastasis (Fig. 1c). To further confirm these results, we detected the expression of CADM2 mRNA and protein in 36 fresh HCC samples and adjacent normal liver tissues, which were collected from Heilongjiang Cancer Hospital. The results showed that CADM2 mRNA and protein expression were significantly lower in HCC tissues than those in adjacent normal liver tissues (Fig. 1d, e). These data demonstrated that CADM2 is significantly down regulated in HCC, deeming that it may serve as a tumor suppressor.

### Overexpression CADM2 inhibits EMT process, migration and invasion of HCC cells

**Fig. 2:**
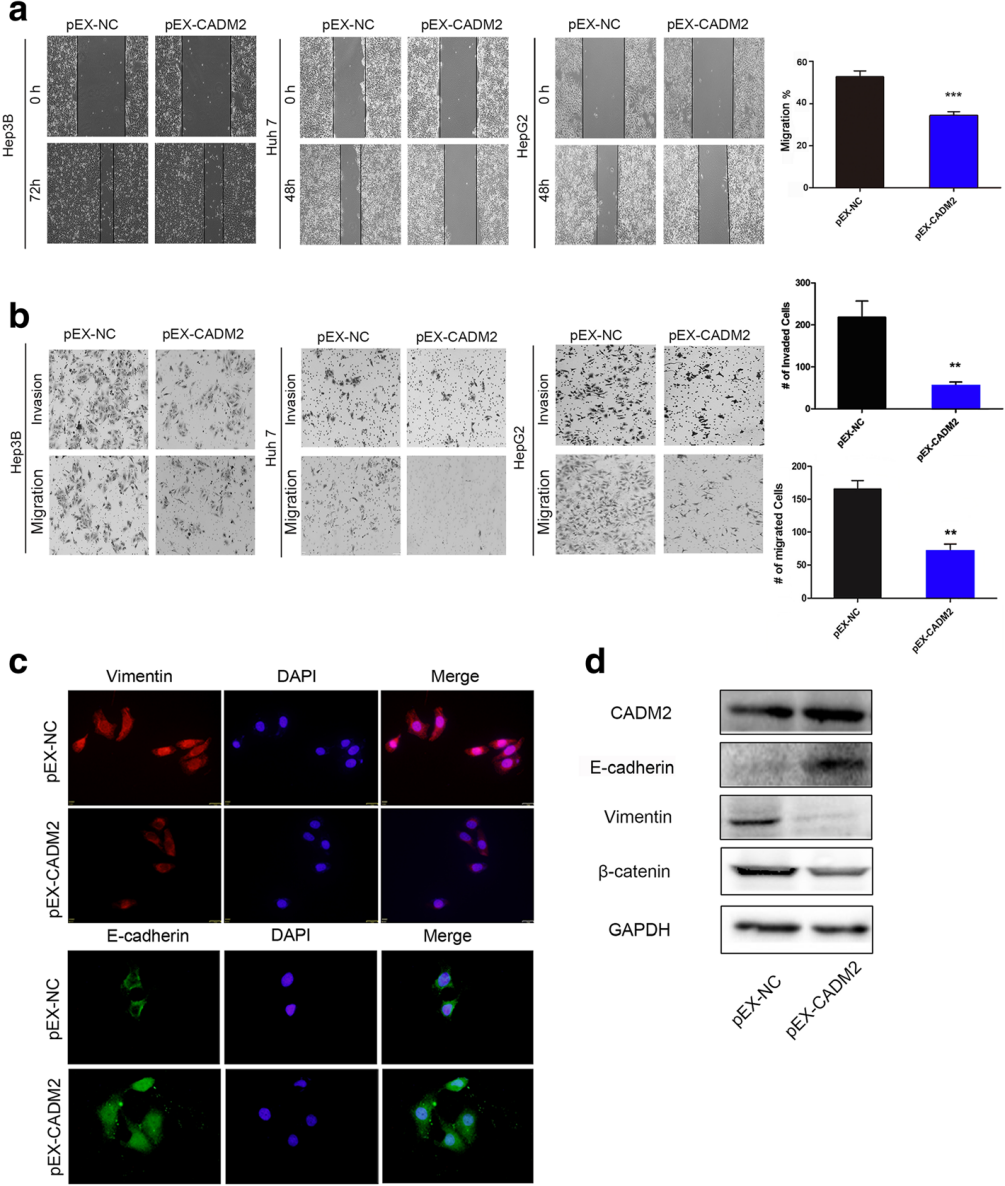
Dysregulated of CADM2 mediated HCC cell migration and invasion. **a**-**b** Representative results of Wound-healing assays (*P* = 0.0008) and Transwell assays (for migration *P* = 0.0054, for invasion *P* = 0.0011) showed the effect of CADM2 expression on the migratory abilities and invasion of HCC cells. Column charts represent mean ± SEM of three independent experiments. Error bars represent SD. (*P < 0.05; **P < 0.01; ***P < 0.001) (**c**). Expression of EMT markers in HepG2 cells transfected with pEX-CADM2 or pEX-NC were examined by immunofluorescence. (Red: Vimentin, Green: E-cadherin, Blue: DAPI) (**d**). Western blotting analysis of indicated proteins in Hep3B cells transfected with pEX-CADM2 or pEX-NC. GAPDH was used as a loading control

Firstly, the expression levels of CADM2 in several liver cell lines were screened. As shown in Fig. 1f, qRT-PCR analysis showed that CADM2 significantly decreased in HCC cell lines compared to normal liver cell line (HL7702). It is worth noting that the expression of CADM2 of Hep3B cells is lowest among these cell lines. To investigate the role of CADM2 in HCC progression, Wound-healing assay and Transwell assay with or without matrigel were performed in HCC cells after transfected with pEX-CADM2 or pEX-NC. The results demonstrated overexpression of CADM2 could dramatically suppress the migratory ability and invasion of HCC cells (Fig. 2a, b). It is believed that EMT process can promote the migratory ability in cancer cells. Next, we examined the EMT markers in HCC cells. Immunofluorescence analysis revealed morphological changes of HCC cells after ectopic expression of CADM2 (Additional file 4: Figure S1). Overexpression of CADM2 resulted in increased expression of E-cadherin (the epithelial cell marker) and decreased expression of Vimentin (the mesenchymal cell marker) (Fig. 2c), thus suggesting that CADM2 could regulate EMT. These results were also confirmed by Western blotting. Meantime, we observed that CADM2 also could inhibit the expression of β-catenin (Fig. 2d). β-catenin also could promote EMT progress [23]. The results demonstrate that overexpression of CADM2 significantly suppresses EMT process and inhibits migratory ability and invasion of HCC cells.

### CADM2 is a direct target of miR-10b

**Fig. 3:**
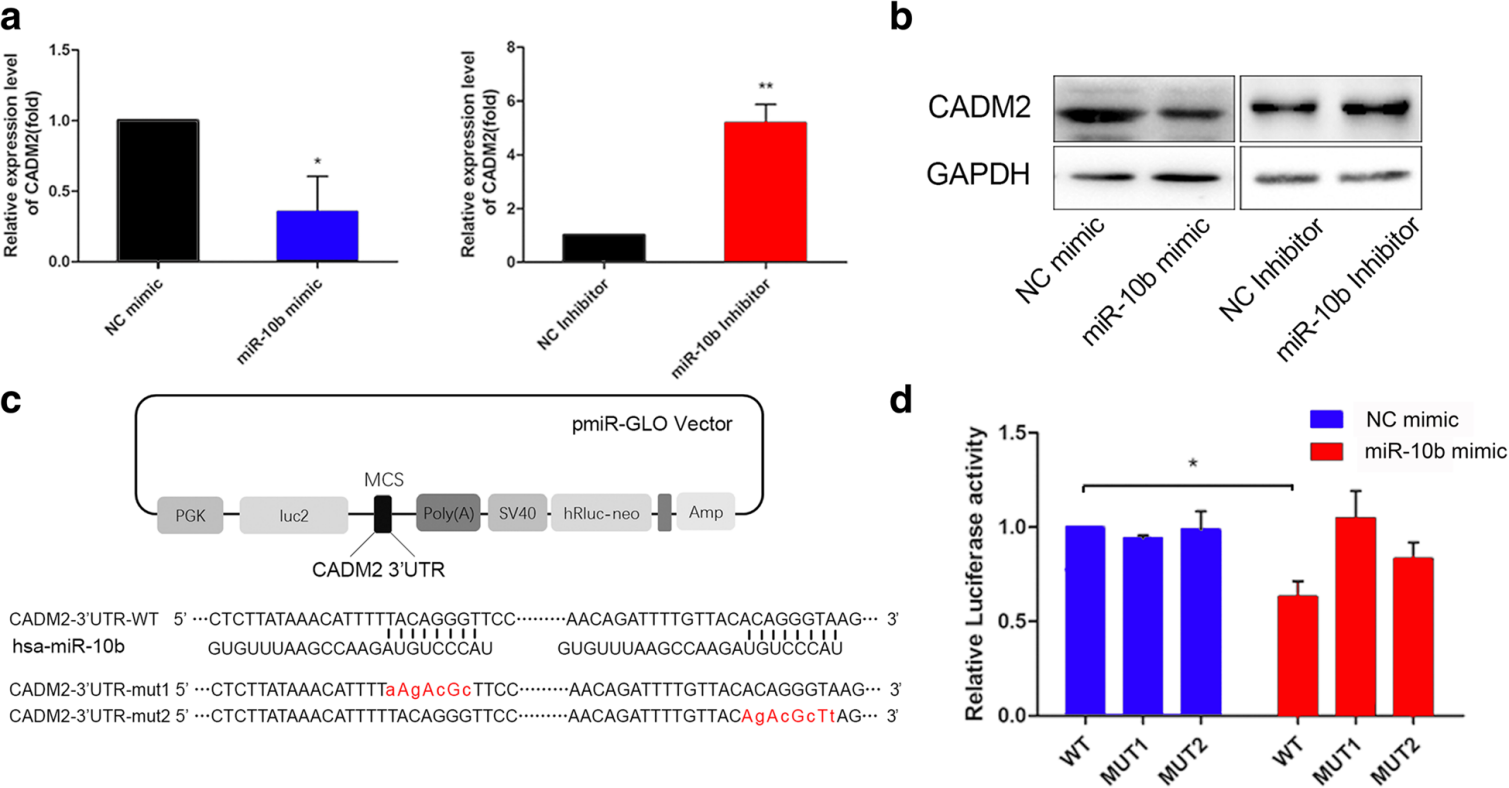
MiR-10b directly targets CADM2 3′-UTR. **a**-**b** The mRNA and protein levels of CADM2 were determined by qRT-PCR and Western blotting after transfection with miR-10b mimic, NC mimic, miR-10b inhibitor, NC inhibitor in HEK293T cells. U6 or GAPDH were used as a loading control. **c** The predicted targeting sites of CADM2 3′-UTR binding to miR-10b were shown. MUT1: the first binding site was mutated; MUT2: the second binding site was mutated). **d** HEK-293T cells were co-transfected with wild-type 3′-UTR (WT) or mutant-type 3′-UTR (MUT1 and MUT2) reporters and NC mimic or miR-10b mimic. In the experiments shown in the panels, luciferase/Renilla activity was measured (*P* = 0.0459). The results are means ± SEM of three independent experiments. (*P < 0.05; **P < 0.01)

We next explored the molecular mechanisms responsible for the metastasis effect of CADM2. miRanda and TargetScan, two bioinformatics algorithms, predicted that CADM2 was a potential target gene of miR-10b. We transfected HEK293T cells with miR-10b mimics and inhibitors followed by detection of mRNA and protein expression of CADM2. We found that miR-10b overexpression led to CADM2 mRNA and protein levels decrease at 72 h post transfection (Fig. 3a, b). To determine whether CADM2 is directly targeted by miR-10b at its 3′-UTR, the luciferase reporter plasmid containing 3′-UTR fragments of CADM2 was co-transfected with miR-10b mimics and NC mimics. There are two predictive miR-10b binding sites in 3′-UTR of CADM2. We built different 3′-UTR fragments including wild type (WT) and mutant-type (MUT1, MUT2) 3′-UTR fragments (Fig. 3c). Luciferase activity assays showed that ectopic expression of miR-10b significantly decreased the luciferase activity of the WT but not that of the MUT1 or MUT2 CADM2 3′ UTR in HEK293T cells (Fig. 3d). These data indicate that miR-10b directly targets the CADM2 3′UTR, thereby reducing CADM2 expression in HCC cells.

### MiR-10b promotes EMT process in HCC cells

**Fig. 4:**
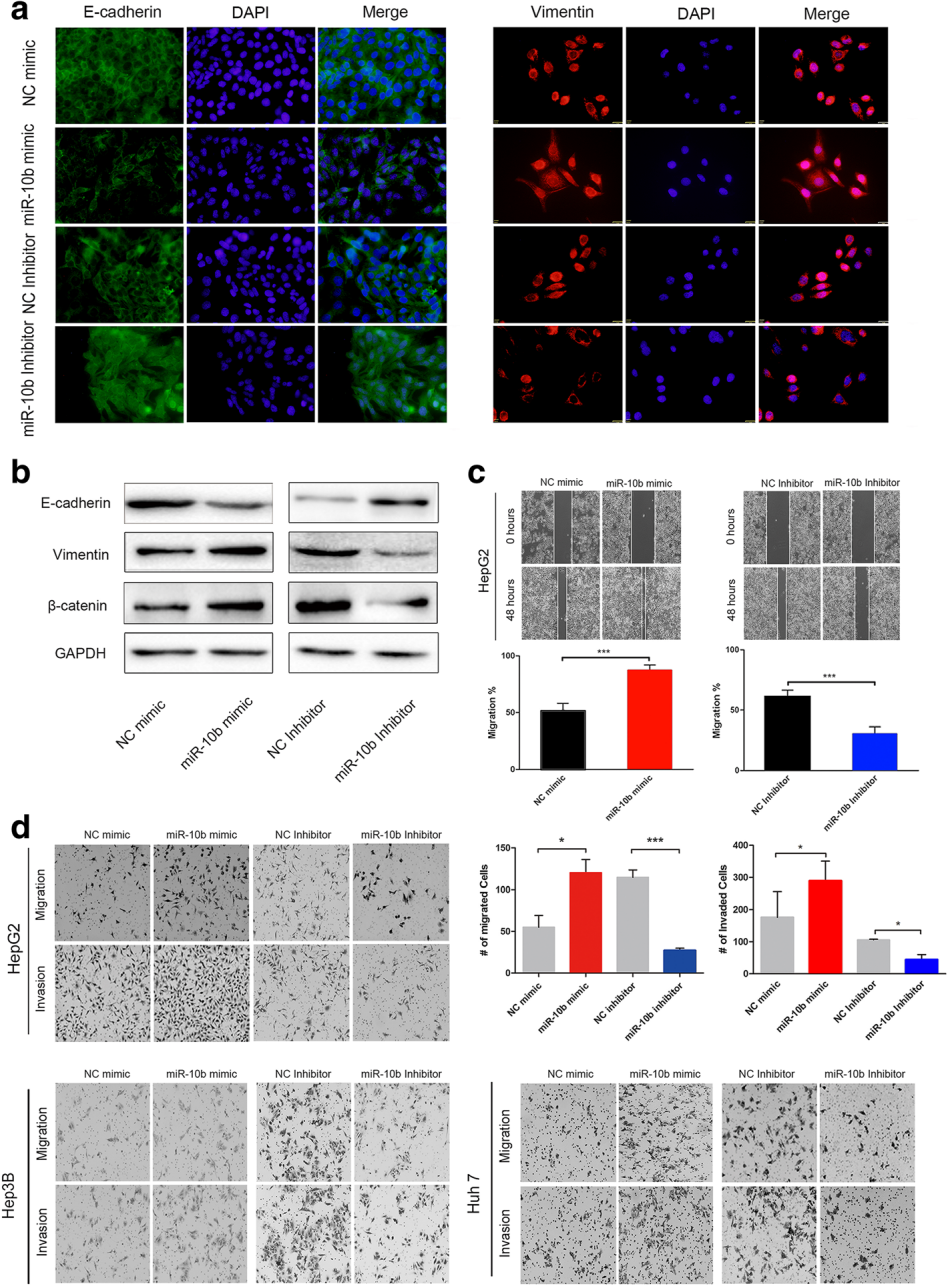
MiR-10b promoted EMT process in HCC cell lines. **a** Expression of EMT markers in HepG2 cells transfected with miR-10b mimic, miR-10b inhibitor or its corresponding control were examined by immunofluorescence. (Green: E-cadherin, Red: Vimentin, Blue: DAPI) (**b**). E-cadherin and Vimentin expression were analyzed by Western blotting in HepG2 cells transfected with miR-10b mimic, miR-10b inhibitor or its corresponding control, GAPDH was used as a loading control. **c** A wound-healing assay was performed using HepG2 cells, and the photos were taken at 0 h and 48 h. **d** The Transwell assays with and without matrigel were performed in three HCC cell lines transfected miR-10b mimic, miR-10b inhibitor or its corresponding control. Representative photos for HepG2, Hep3B, Huh7 cells and quantitative analysis only for HepG2 are shown. Data represents mean ± SEM of three independent experiments. Error bars represent SD. (*P < 0.05; **P < 0.01; ***P < 0.001)

Recent publications strongly support that miR-10b plays a vital role in the development of a variety of tumors, especially metastasis [24, 25]. Several studies find that miR-10b is able to promote invasion and migration as well as regulate EMT process in several tumor cells [26–28]. However, there is no report on whether miR-10b can regulate EMT in HCC. To investigate whether miR-10b could regulate EMT process in HCC cells, we altered the expression of miR-10b through transient transfection of HCC cells with a miR-10b mimic or inhibitor. The expression of miR-10b in HCC cells was confirmed by qRT-PCR after transfection for 48 h (Additional file 5: Figure S2). In Immunofluorescence assay, overexpression of miR-10b inhibited E-cadherin expression and increased the Vimentin expression (Fig. 4a). To further confirm that miR-10b could promote the EMT of HCC cells, we examined the expression levels of E-cadherin and Vimentin in HCC cell lines by Western blotting (Fig. 4b).

A wound-healing assay and Transwell assay with or without matrigel showed that overexpression of miR-10b could markedly promote the migratory ability and invasion of HCC cells. Meanwhile, silencing of miR-10b suppressed the migratory ability and invasion of HCC cells (Fig. 4c, d). These results demonstrate that overexpression of miR-10b significantly promotes cell migration and invasion as well as EMT in HCC cell lines.

### Correlation between CADM2 and miR-10b in human HCC samples

**Fig. 5:**
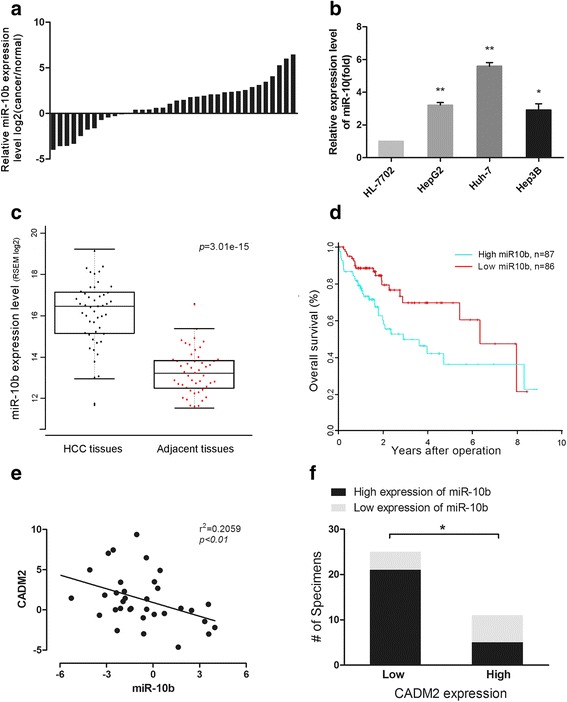
Correlation between CADM2 and miR-10b in human HCC samples. **a** Relative miR-10b expression levels in HCC samples. **b** qRT-PCR for detecting miR-10b expression levels in several HCC cell lines and normal liver cell line as indicted. **c** The expression of miR-10b in cancerous tissues and matched normal tissues from TCGA (P = 3.01e-15). **d** Kaplan-Meier analysis of over-all survival of patients with different miR-10b expression level (*P* = 0.0127). **e** The Spearman’s correlation analysis between miR-10b expression and CADM2 expression in HCC samples (*P* = 0.0054). **f** The relationship between miR-10b expression and CADM2 protein expression was analyzed using Fisher exact test (*P* = 0.039). Data represents mean ± SEM of three independent experiments. Error bars represent SD. (*P < 0.05; **P < 0.01)

In order to validate the correlation between CADM2 and miR-10b expression in human HCC tissues, we firstly collected and analyzed fresh human HCC samples and corresponding non-tumor tissues from HCC resection. In the analysis of primary tumor specimens from 36 patients with HCC by qRT-PCR, miR-10b expression was found to be significantly higher in tumor tissues compared to corresponding non-tumor tissues (Fig. 5a). In addition, the level of miR-10b in liver cell lines also was detected. As we expected, the expression of miR-10b is higher in HCC cells compared to that in normal liver cells (HL7702) (Fig. 5b). Further more, higher expression of miR-10b in HCC samples was also observed in high-throughput sequencing data from TCGA compared with normal tissues (Fig. 5c). Kaplan-Meier analysis indicates that high expression of miR-10b is negatively correlated with the overall survival time of HCC patients (Fig. 5d).

We used qRT-PCR to measure CAMD2 expression in the same cohort that was used to examine the expression level of miR-10b. There was an inverse correlation between miR-10b and CADM2 expression (Fig. 5e, r^2^ = 0.2059, *P* = 0.0054). Moreover, 36 HCC samples were grouped into low expression (*n* = 24) and high expression (*n* = 12) according to CADM2 expression. In the low CADM2 expression group, 21/24 of the samples showed high level of miR-10b (Fig. 5f, Additional file 6: Table S2). The results show that there is a significant negative correlation between miR-10b and CADM2 expression levels in HCC samples.

### FAK/AKT pathway mediates the effect of miR-10b/CADM2 in HCC metastasis

**Fig. 6:**
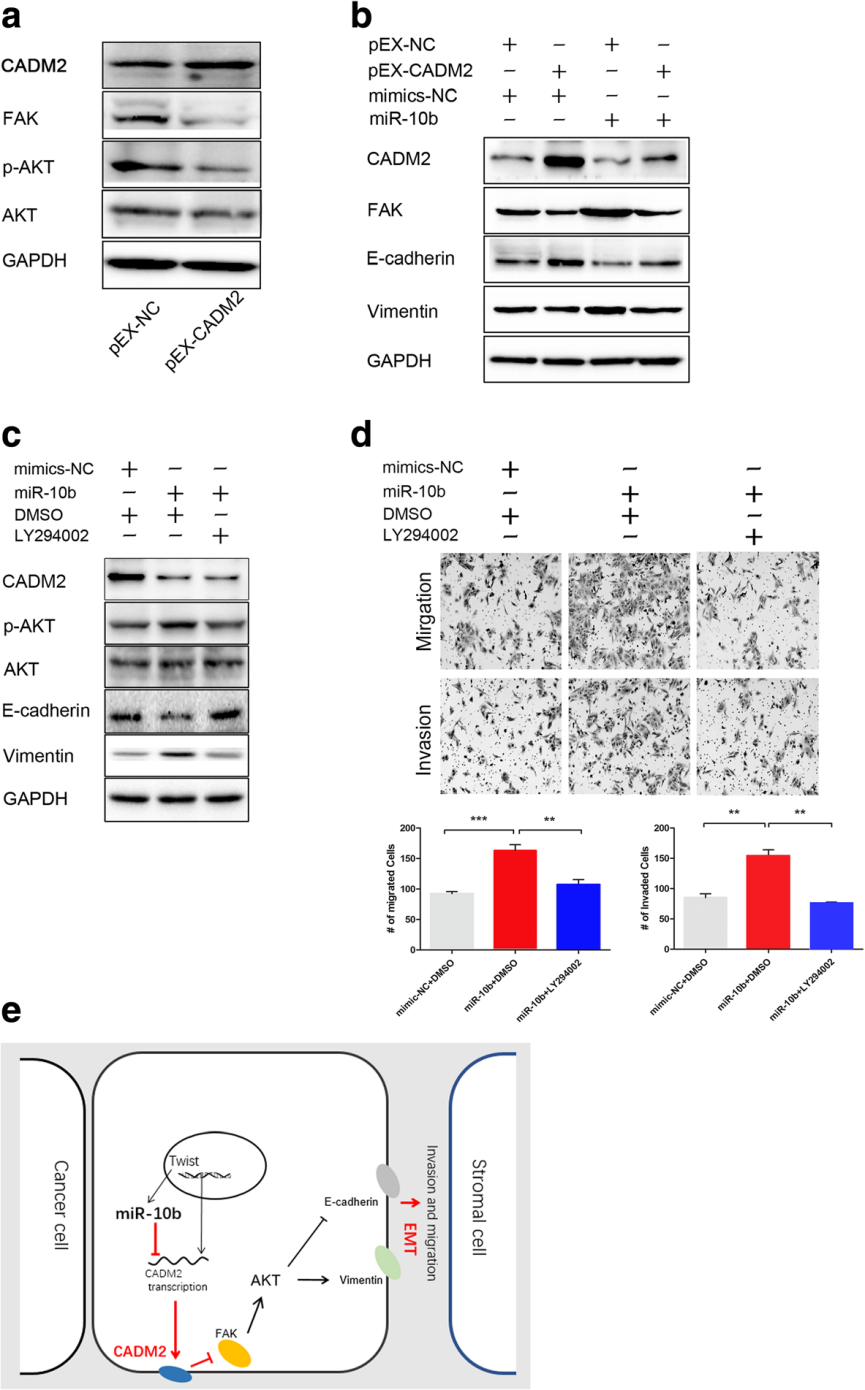
FAK/AKT pathway mediates the effect of miR-10b/CADM2 in HCC metastasis. **a** Western blotting analysis of indicated proteins in Hep3B cells transfected with pEX-CADM2 or pEX-NC. **b** Hep3B cells were co-transfected with miR-10b mimic (or NC mimic) and pEX-CADM2 (or pEX-NC). The expression of CADM2, FAK and EMT markers were detected by Western blotting. **c** Treatment with LY294002 reversed EMT mediated by miR-10b overexpression. GAPDH was used as a loading control. **d** The cells transfected as in Fig. 6c were used to perform Transwell assays. **e** Schematic representation of CADM2 actions in HCCs. Data represents mean ± SEM of three independent experiments. Error bars represent SD. (*P < 0.05; **P < 0.01; ***P < 0.001)

To investigate the underlying mechanisms on how miR-10b/CADM2 modulates HCC metastasis. We found out that overexpression of CADM2 suppressed the expression of FAK and p-AKT in Hep3B cells (Fig. 6a). MiR-10b overexpression increased FAK level and activated AKT pathway (Fig. 6b, c). Co-transfection miR-10b with CAMD2 partially rescued the changes of EMT markers and FAK induced by miR-10b overexpression (Fig. 6b). To further confirm miR-10b/CADM2 regulates EMT process via regulating AKT pathway, we treated HCC cells with a PI3K inhibitor, LY294002 (20 μmol/L) to block PI3K/AKT signaling. Then Western blotting and Transwell assay were performed. The results demonstrated that LY294002 partially reversed EMT process and increased migratory and invasion ability induced by miR-10b (Fig. 6c, d). These data indicated that miR-10b could activate FAK/AKT pathway to promote EMT process through inhibiting its target gene CADM2 (Fig. 6e).

## Discussion

Hypo-expression of CADM2 gene expression has been observed in prostate cancer [18], ovarian cancer [29], lymphoma [30], melanoma [31] and liver cancer [20]. Previous studies also suggest that CADM2 might be involved in the maintenance of cell polarity and adhesion. Disruption of cell adhesion in the primary tumor is an initial step of cancer invasion and metastasis [32]. In this study, we reported that CADM2 serves as a tumor suppressor that negatively control HCC metastasis. Clinical sample analysis verified that CADM2 was downregulated in HCC tissues compared to normal liver tissues. Furthermore, CADM2 was a direct target of miR-10b, which modulates the FAK/AKT signaling pathway to suppress migration, invasion as well as EMT process in HCC.

Previous studies have pointed out that microRNA plays a major role in development and progression of several cancers including HCC [33–36]. miRNAs play an important role in regulating gene expression as a manner of posttranscriptional modification. During exploring the mechanism of CADM2 regulation, we found out that CADM2 might be potential target gene of miR-10b. Fortunately, our results support this hypothesis. The role of miR-10b in cancer is studied earlier. And accumulating evidences suggest that miR-10b is an important Onco-miR and elevated miR-10b has been detected in several tumors including HCC. Our results demonstrated that miR-10b regulation is one of the reasons of CADM2 downregulation in HCC. Meantime, we also noticed several studies verify that promoter methylation of CADM2 is one of reasons which lead to hypo-expression of CADM2 in tumor tissues [18]. Therefore, we also analyzed promoter methylation of CADM2 in liver cancer tissues using TCGA data. The results suggested that epigenetic modification might be one of the reasons for hypo-expression of CADM2 in HCC (Additional file 7: Figure S3). But promoter methylation change of CADM2 in HCC needs further experiments.

FAK is a focal adhesion-associated protein kinase involved in cellular adhesion and spreading processes. It is a critical mediator that connects integrin and the downstream signal molecules in integrin-signal transduction pathway, and is the convergence of many signal pathways [37–40]. Accumulated evidences indicate that FAK is overexpressed in several cancers and promotes cancer progression and metastasis. Both PI3K/AKT and ERK signaling pathways are important downstream effectors of FAK [40], and contribute to EMT, invasion and metastasis in cancers [41, 42]. In contrast, overexpression of CADM2 decreased the activity of FAK and AKT (Fig. 6a). However, overexpression of CADM2 has a very limited effect on the activity of MEK and ERK in HCC cells (Data not shown). As expected, our results demonstrated that miR-10b increased AKT phosphorylation, whereas CADM2 overexpression has the reverse effect. Furthermore, the inactivation of the PI3K/AKT pathway abolished enhance of EMT, migration and invasion due to miR-10b overexpression in HCC (Fig. 6c, d).

Taken together, our results provide new evidence, which CADM2 acts as a tumor suppressor gene in HCC. The newly identified miR-10b-CADM2-FAK/AKT axis provides a new insight into the development of HCC, especially with respect to migration and invasion, and represents us with a new, potential therapeutic target for HCC treatment.

## Conclusions

This study concludes that CADM2, as a new target of miR-10b, inhibits EMT, migration and invasion of HCC cell through FAK/AKT pathway. The study recognizes CADM2 might be a useful biomarker for metastasis prediction of HCC patients. This finding may broaden understanding of mechanisms involved in cancer metastasis and suggest novel targets for HCC treatment.

## Additional files

Additional file 1: **Table S1.** Clinical and pathological characteristics of 36 HCC patients. (DOCX 29 kb)

Additional file 2: **Table S3.** Primers and RNA oligonucleotides. (DOCX 22 kb)

Additional file 3: **Table S4.** Information of antibodies used in this study. (DOCX 18 kb)

Additional file 4: **Figure S1.** Overexpression CADM2 inhibits EMT process in HCC cells. (JPG 4823 kb)

Additional file 5: **Figure S2.** Transfection efficiency in HCC cells. (JPG 6942 kb)

Additional file 6: **Table S2.** Correlations between clinical features and CADM2 expression in 36 HCC patients. (DOCX 19 kb)

Additional file 7: **Figure S3.** Correlation between CADM2 mRNA and its DNA methylation level in human HCC samples. (JPG 7198 kb)

## Abbreviations

3’-UTR: 3’-Untranslated Region
CADM2: Cell Adhesion Molecule 2
DMSO: Dimethyl Sulfoxide
EMT: Epithelial–Mesenchymal Transition
FAK: Focal Adhesion Kinase
HCC: Hepatocellular Carcinoma
IF: Immunofluorescence
miRNAs: microRNAs
qRT-PCR: Real-time Quantitative Polymerase Chain Reaction
