# Supplementary material for: CADM2, as a new target of miR-10b, promotes tumor metastasis through FAK/AKT pathway in hepatocellular carcinoma
Source: J Exp Clin Cancer Res. 2018 Mar 5;37:46. doi: 10.1186/s13046-018-0699-1 (PMC5836378; doi:10.1186/s13046-018-0699-1)
Supplement: Supplementary file 1 — Table S1. Clinical and pathological characteristics of 36 HCC patients. (DOCX 29 kb) [file 13046_2018_699_MOESM1_ESM.docx]

**Additional file 1: Table S1.** Clinical and pathological characteristics of 36 HCC patients.

| Sample ID | Age | Gender | Tumor Size (>5cm) | Treatment | Liver cirrhosis | Hepatitis Type | Pathological Differentiation Grade |
| --- | --- | --- | --- | --- | --- | --- | --- |
| 554762 | 53 | F | Y | Operation | Y | HBV | Advanced |
| 633763 | 47 | F | N | Operation | N | No | Moderate-Advanced |
| 633919 | 41 | F | N | Operation | N | No | Advanced |
| 634506 | 62 | M | N | Operation | N | HBV | Moderate-Advanced |
| 635807 | 50 | M | N | Operation | Y | HBV | Moderate |
| 637984 | 64 | M | Y | Operation | Y | HBV | Moderate-Advanced |
| 638216 | 63 | F | Y | Operation | N | HCV | Moderate |
| 642431 | 46 | M | Y | Operation | Y | HBV | Moderate |
| 642568 | 44 | M | N | Operation | Y | HBV | Moderate |
| 642685 | 56 | M | Y | Operation | Y | HBV | Moderate |
| 642808 | 65 | M | Y | Operation | Y | HBV | Moderate |
| 643073 | 42 | M | Y | Operation | N | HBV | Moderate |
| 643079 | 55 | M | Y | Operation | N | HBV | Moderate-Advanced |
| 643376 | 48 | M | Y | Operation | N | No | Moderate |
| 643391 | 69 | M | Y | Operation | Y | HBV | Moderate |
| 643452 | 47 | M | Y | Operation | Y | HBV | Moderate |
| 643814 | 64 | M | Y | Operation | Y | HBV | Moderate |
| 643933 | 67 | M | Y | Operation | Y | HBV | Moderate-Low |
| 648475 | 61 | F | Y | Operation | N | HBV | Moderate |
| 649603 | 53 | M | Y | Operation | Y | HBV | Moderate-Low |
| 649727 | 46 | M | Y | Operation | Y | HBV | Moderate |
| 653366 | 44 | M | Y | Operation | Y | HBV | Moderate |
| 653368 | 48 | F | Y | Operation | N | HBV | Moderate |
| 656355 | 36 | M | Y | Operation | Y | HBV | Moderate-Low |
| 656361 | 52 | M | N | Operation | Y | HBV | Moderate-Low |
| 662800 | 53 | F | Y | Operation | N | No | Moderate |
| 663148 | 65 | M | Y | Operation | N | HBV | Moderate-Low |
| 663895 | 46 | M | Y | Operation | Y | HBV | Moderate-Advanced |
| 663976 | 48 | F | Y | Operation | N | HBV | Moderate |
| 663998 | 65 | M | Y | Operation | Y | HBV | Moderate |
| 664353 | 70 | M | Y | Operation | Y | HBV | Moderate-Advanced |
| 664515 | 47 | M | Y | Operation | Y | HBV | Moderate-Advanced |
| 664527 | 50 | M | Y | Operation | Y | HBV | Moderate-Advanced |
| 676274 | 64 | M | Y | Operation | Y | HBV | Moderate-Advanced |
| 676859 | 45 | M | Y | Operation | Y | HBV | Moderate-Advanced |
| 677044 | 65 | M | Y | Operation | N | No | Moderate |
