# Supplementary material for: CADM2, as a new target of miR-10b, promotes tumor metastasis through FAK/AKT pathway in hepatocellular carcinoma
Source: J Exp Clin Cancer Res. 2018 Mar 5;37:46. doi: 10.1186/s13046-018-0699-1 (PMC5836378; doi:10.1186/s13046-018-0699-1)
Supplement: Supplementary file 2 — Table S3. Primers and RNA oligonucleotides. (DOCX 22 kb) [file 13046_2018_699_MOESM2_ESM.docx]

**Additional file 2: Table S3.** Primers and RNA oligonucleotides.

| Reverse transcription | |
| --- | --- |
| miR-10b stem-loop | GTCGTATCCAGTGCGTGTCGTGGAGTCGGCAATTGCACTGGATACGACCACAAA |
| U6 | CGCTTCACGAATTTGCGTGTCAT |
| qRT-PCR | |
| miR-10b F | TACCCTGTAGAACCGA |
| miR-10b R | TGGAGTCGGCAATTGCA |
| U6 F | GCTTCGGCAGCACATATACTAAAAT |
| U6 R | CGCTTCACGAATTTGCGTGTCAT |
| CADM2 F | TCTATTCCAACAAGTCAGAAAATAATG |
| CADM2 R | CGCTTAGACTTGATTTTGACGG |
| GAPDH F | GGAGCGAGATCCCTCCAAAAT |
| GAPDH R | GGCTGTTGTCATACTTCTCATGG |
| FAK F | TGGTGCAATGGAGCGAGTATT |
| FAK R | CAGTGAACCTCCTCTGACCG |
| PCR | |
| CADM2 3’UTR F | AACGCAGGTGTTCCCAGT |
| CADM2 3’UTR R | GGTCTAACACAAATATGAGACCC |
| MUT F | ATACATGCTCTGGGTTTTGA |
| MUT R | TCAAAACCCAGAGCATGTAT |
| CADM2 3’UTR-mut-1F | GTGCAAATGGGTGCGTCTTAAAATGTTTATAAG |
| CADM2 3’UTR-mut-1R | ATAAACATTTTAAGACGCTTCCCATTTGC |
| CADM2 3’UTR-mut-2F | GCCTTTGGCTAAGCGTCTGTAACAAAATC |
| CADM2 3’UTR-mut-2R | CAGATTTTGTTAAGACGCTTAGCCAAAGGCT |
| RNA oligonucleotide | |
| miR-10b mimic | UACCCUGUAGAACCGAAUUUGUG/CAAAUUCGGUUCUACAGGGUAUU |
| NC mimic | UUCUCCGAACGUGUCACGUTT/ ACGUGACACGUUCGGAGAATT |
| miR-10b inhibitor | CACAAAUUCGGUUCUACAGGGUA |
| NC inhibitor | UUGUACUACACAAAAGUACUG |
