# Supplementary material for: CADM2, as a new target of miR-10b, promotes tumor metastasis through FAK/AKT pathway in hepatocellular carcinoma
Source: J Exp Clin Cancer Res. 2018 Mar 5;37:46. doi: 10.1186/s13046-018-0699-1 (PMC5836378; doi:10.1186/s13046-018-0699-1)
Supplement: Supplementary file 3 — Table S4. Information of antibodies used in this study. (DOCX 18 kb) [file 13046_2018_699_MOESM3_ESM.docx]

**Additional file 3: Table S4.** Informationof antibodies used in this study

| Antibody | WB | IHC | Specificity | Company |
| --- | --- | --- | --- | --- |
| CADM2 | 1:500 | 1:100 | Rabbit  polyclonal | Abcam |
| E-cadherin (#14472) | 1:1000 | 1:50 | Mouse monoclonal | Cell Signaling Technology |
| Vimentin (#5741) | 1:1000 | 1:100 | Rabbit  polyclonal | Cell Signaling Technology |
| AKT (#4685) | 1:1000 | - | Rabbit monoclonal | Cell Signaling Technology |
| p-AKT (#4060) | 1:1000 | - | Rabbit  monoclonal | Cell Signaling  Technology |
| FAK (#wl01696) | 1:1000 | - | Rabbit monoclonal | WanLei |
| MEK | 1:1000 | - | Rabbit monoclonal | Cell Signaling Technology |
| p-ERK (#4370) | 1:1000 | - | Rabbit monoclonal | Cell Signaling Technology |
| ERK (#4695) | 1:1000 | - | Rabbit monoclonal | Cell Signaling Technology |
| GAPDH (ab181602) | 1:10000 | - | Rabbit monoclonal | Abcam |
