# Supplementary figures and images for: CADM2, as a new target of miR-10b, promotes tumor metastasis through FAK/AKT pathway in hepatocellular carcinoma
Source: J Exp Clin Cancer Res. 2018 Mar 5;37:46. doi: 10.1186/s13046-018-0699-1 (PMC5836378; doi:10.1186/s13046-018-0699-1)

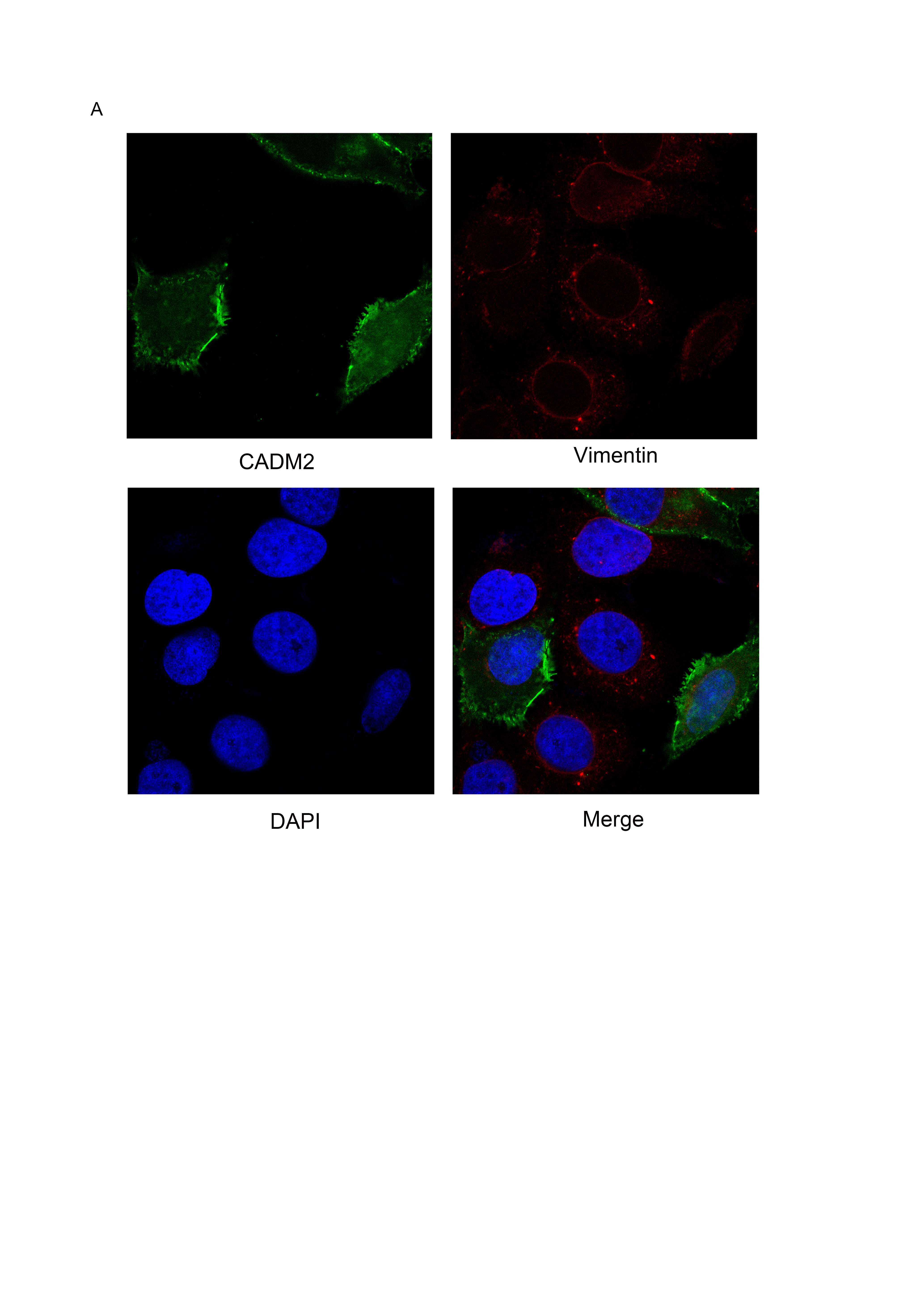

Supplement: Supplementary file 4 — Figure S1. Overexpression CADM2 inhibits EMT process in HCC cells. (JPG 4823 kb) [file 13046_2018_699_MOESM4_ESM.jpg]
