# Supplementary material for: CADM2, as a new target of miR-10b, promotes tumor metastasis through FAK/AKT pathway in hepatocellular carcinoma
Source: J Exp Clin Cancer Res. 2018 Mar 5;37:46. doi: 10.1186/s13046-018-0699-1 (PMC5836378; doi:10.1186/s13046-018-0699-1)
Supplement: Supplementary file 6 — Table S2. Correlations between clinical features and CADM2 expression in 36 HCC patients. (DOCX 19 kb) [file 13046_2018_699_MOESM6_ESM.docx]

| Clinical features | n | CADM2 expression | |
| --- | --- | --- | --- |
| Variables |  | low | high |
| **Gender** | 36 |  |  |
| Male | 28 | 19 | 9 |
| Female | 8 | 6 | 2 |
| **Age (years)** |  |  |  |
| ≤ 60 | 23 | 15 | 8 |
| > 60 | 13 | 10 | 3 |
| **Liver cirrhosis** |  |  |  |
| Presence | 23 | 16 | 7 |
| Absence | 13 | 9 | 4 |
| **Hepatitis** |  |  |  |
| Presence | 31 | 23 | 8 |
| Absence | 5 | 2 | 3 |
| **Tumor size** (cm) |  |  |  |
| ≤ 5cm | 6 | 4 | 2 |
| > 5cm | 30 | 19 | 11 |
| **Differentiation** |  |  |  |
| Advanced | 12 | 11 | 1 |
| Moderate | 19 | 10 | 9 |
| Low | 5 | 4 | 1 |

**Additional file 6: Table S2.** Correlations between clinical features and CADM2 expression in 36 HCC patients
